# Host factors influencing susceptibility to HIV infection and AIDS progression

**DOI:** 10.1186/1742-4690-4-52

**Published:** 2007-07-25

**Authors:** Juan Lama, Vicente Planelles

**Affiliations:** 1 La Jolla Institute for Molecular Medicine, 4570 Executive Drive, Suite 100, San Diego, California 92121, USA; 2 RetroVirox, Inc. 4570 Executive Drive, Suite 100, San Diego, California 92121, USA; 3 Department of Pathology, University of Utah School of Medicine, 15 North Medical Drive East #2100 – Room 2520, Salt Lake City, Utah 84112, USA

## Abstract

Transmission of HIV first results in an acute infection, followed by an apparently asymptomatic period that averages ten years. In the absence of antiretroviral treatment, most patients progress into a generalized immune dysfunction that culminates in death. The length of the asymptomatic period varies, and in rare cases infected individuals never progress to AIDS. Other individuals whose behavioral traits put them at high-risk of HIV transmission, surprisingly appear resistant and never succumb to infection. These unique cases highlight the fact that susceptibility to HIV infection and progression to disease are complex traits modulated by environmental and genetic factors. Recent evidence has indicated that natural variations in host genes can influence the outcome of HIV infection and its transmission. In this review we summarize the available literature on the roles of cellular factors and their genetic variation in modulating HIV infection and disease progression.

## Background

The period of asymptomatic disease after HIV-1 infection averages about ten years, although it may vary greatly among infected subjects [1]. The existence of attenuated viral strains that fail to induce disease in animal models has long been known. Similarly, it is now widely accepted that human allelic variants for certain genes can influence the susceptibility to HIV-1 infection [2,3]. Supporting a role for genetic factors in the host, several studies have shown that susceptibility to HIV-1 in vitro largely varies among individual donors. Conversely, primary cells from homozygotic twins display much less variation in their permissivity to infection [4-8].

Like all viruses, HIV-1 must usurp the cellular machinery at multiple steps to complete a productive cycle. The virus enters cells by fusing with the cellular membrane, taking advantage of receptor and co-receptor host proteins, which otherwise play important roles in immunity and inflammation. Then, the viral genetic material is delivered into the cytoplasm in the form of a nucleoprotein core. The viral RNA genome is copied into DNA, transported to the cell nucleus, and integrated in the host chromosome. The proviral HIV-1 DNA is transcribed into viral mRNAs, which are then processed and exported to the cytoplasm. Upon translation, viral products are transported to budding sites where virions are assembled together with viral RNA. For each of these steps, HIV-1 relies on cellular proteins. Only a fraction of these host proteins have been identified, but their role in the HIV-1 life cycle is currently a subject of intense investigation.

### Approaches to study HIV disease progression

Several approaches have been used to study HIV pathogenesis in vivo. The availability of non-human primate models has largely advanced our understanding of the field. Studies with animal models have highlighted the importance of the so-called viral "accessory genes" in HIV disease progression. These genes were initially deemed non-essential in in vitro studies because the virus would be able to replicate despite their removal from the viral genome [9]. Despite the usefulness of animal models to define viral determinants of pathogenesis, the genetic differences between human and non-human primates, have made the latter less amenable for the study the role of host factors.

Long-term nonprogressors (LTNP) have provided a unique opportunity to study the mechanisms of HIV disease. LTNPs are HIV-infected individuals who have lived free of symptoms for extended periods of time, in the absence of antiretroviral treatment. A standard criterion for LTNP status is to have had a documented infection for ten years or more, stable CD4-positive T cell counts above 500 cells/ml, and plasma viral load below 10,000 RNA copies/ml. Depending on the definition of "nonprogression" used, this population has been estimated to represent 2–4% of all infected patients [10]. The recruitment of LTNP cohorts is a formidable task, because until recently, most patients with well documented clinical histories had been treated before the onset of symptoms.

An additional approach to examine disease progression is to investigate highly exposed uninfected (EU) individuals. EUs are subjects who resist HIV infection and seroconversion, despite being at high-risk for transmission. EU cohorts have been gathered from groups of intravenous drug users (IDU), sex workers, children born to seropositive mothers, individuals performing unprotected sex with multiple partners, and health care workers undergoing accidental exposure to the virus [11].

Important insight into HIV pathogenesis can also be gained by studying the natural course of infection in seropositive patients. Clinical variables (decline in CD4 counts, increase in viral load) have been used to monitor the rate of progression to disease in untreated patients, or to establish prognosis in terms of virologic and immunologic success in patients following antiretroviral regimes. These variables can be statistically associated with host genotypic variants or specific phenotypic traits.

Finally, the study of healthy HIV-seronegative patients who may bear genetic markers of interest, can also shed light into the mechanisms of HIV pathogenesis. The role of cellular factors influencing HIV replication and immunity can be addressed by exposing primary cells from healthy seronegative individuals to virus in vitro. Likewise, statistical associations between haplotypes or single-nucleotide polymorphisms (SNP) can be drawn by monitoring the extent of viral replication in vitro. When available, genetic associations with the rate of replication in these ex-vivo models can also be validated with in vivo data monitoring disease progression [12].

Factors influencing susceptibility to infection and the course of disease can be grouped into three categories: 1) viral factors determining the replicative properties of the virus or its ability to escape immune responses; 2) cellular factors modulating the innate or acquired immune responses to infection; and 3) cellular factors co-operating with viral products that govern the ability of the virus to replicate in human cells. Thus, the rate at which an untreated HIV-infected patient progresses to AIDS may be explained by a combination of these factors, which ultimately dictate how fast HIV replicates and/or how efficiently it overcomes the immune defenses posed by the host. Below, we will discuss cellular factors influencing various steps of the HIV life cycle, and those modulating the innate immune response. The role of the HLA system in AIDS progression is beyond the scope of this review, and has been amply discussed in other reviews [13-15].

## Host factors modulating viral entry

### The HIV-1 co-receptors: CCR5 and CXCR4

Entry into target cells occurs by a multi-step process that culminates with the fusion of viral and cellular membranes. HIV-1 utilizes CD4 as its primary receptor. Binding to CD4 is followed by conformational changes in the viral envelope that lead to the engagement of one of the viral co-receptors (CCR5 or CXCR4) [16]. Based on their functionality in vitro, other chemokine receptors may also work as HIV-1 co-receptors. Among them are CCR2, CCR3, CCR8, CCR9, CXCR6, CX3CR1, ChemR23, APJ, and RDC1. However, CCR5 and CXCR4 constitute the major co-receptors in vivo (for a recent review see [17]).

CXCR4 was the first co-receptor identified [18]. Afterwards, the role of CCR5 in HIV-1 infection was soon elucidated [19-23]. Soon after these findings, researchers sought genetic polymorphisms that could confer protection to HIV-1 infection [19-21,23,24]. These studies characterized the CCR5Δ32 allele, which has been unequivocally associated with protection to HIV-1 infection in homozygotic individuals [24-26]. This discovery provided the first conclusive evidence for the existence of genetic resistance to HIV-1 infection. CCR5Δ32 expresses a truncated co-receptor that is not transported to the cell surface and thus is incompetent for viral entry [27]. Individuals homozygous for the Δ32 allele seem to have a normal life expectancy, although immunological differences have been reported, which may influence the outcome of infections with other pathogens, such as West Nile and hepatitis C virus (reviewed in [17,28]). The apparent lack of immunological dysfunction in individuals with the homozygousΔ32 genotype may be explained by the functional redundancy in the chemokine receptors and their ligands. The CCR5Δ32 allele occurs at a frequency of 4–15% in the Caucasian population, with higher frequencies in Northern European populations. However, the CCR5Δ32 allele is rarely found in Asians and Africans. Approximately 1% of Caucasians carry two copies of the Δ32 allele [29]. These individuals are overrepresented in cohorts of high-risk HIV-seronegative individuals (EUs) [25,30,31]. Protection against HIV-1 infection in homozygous CCR5Δ32 individuals, however, is not complete. Although rare, infections of homozygous CCR5Δ32 have been reported, but always in patients infected with virus strains utilizing the CXCR4 co-receptor [32-37]. Other studies have reported increased frequencies of CCR5Δ32 heterozygotes among LTNP, and in patients progressing to disease at slower than normal rates [38-41]. However, these associations have not been observed in all cohorts, suggesting that the CCR5Δ32 allele alone may not universally slow progression to AIDS [42-45]. Genetic differences among the ethnicities evaluated, or different transmission routes in the studied cohorts may explain these discrepancies.

The observation that high level of CCR5 expression on CD4-positive primary T cells is associated with high viral loads and accelerated disease progression further highlights the contribution of CCR5 to disease progression [46,47]. Generally, lymphocytes from Δ32 heterozygous individuals express lower surface levels of CCR5, as compared to those observed on cells from individuals homozygous for the wild type gene [48]. In the previous CCR5 expression in heterozygous individuals was lower than the expected 50%, relative to wild-type homozygous. This observation led the authors to hypothesize that the truncated co-receptor may dimerize with the full-length protein and interfere with its transport.

Other mutations in the CCR5 coding region have been described. Some of them introduce frame-shifts that result in truncated proteins that, similarly to the Δ32 variant, fail to be transported to the cell surface (e.g., FS299). Other mutations (e.g. C20S and C269F) affect the formation of disulfide bridges, altering surface expression of the receptor and the ability to bind ligands [49]. Some mutations result in undetectable or very low levels of surface receptor (C269F, G106R, C101X). Unlike Δ32, most of these CCR5 variants are relatively rare (frequencies below 1–2%) and only present in specific populations. Thus, their role in HIV-1 disease progression has not been properly established [17,49,50]. Interestingly, the rare C20S, C101X (also called m303), and T303A alleles are over-represented in EU individuals also carrying the more common CCR5Δ32 allele [51,52]. These findings suggest that alleles that may, in the context of a wild type allele, have a weak effect, may exert a more profound protection in combination with CCR5Δ32.

Genetic variants have also been described in the CCR5 promoter. Most changes are single base substitutions that could potentially alter the level of expression. CCR5P1, one of the multi-site haplotypes identified in the CCR5 promoter, is composed of 13 distinct SNPs. This haplotype has been associated with faster progression to AIDS in individuals carrying the wild type coding region for CCR5 and CCR2, and homozygous for CCR5P1 [53]. A similar association with rapid progression has been reported for an A/G polymorphism located in the first CCR5 intron. Individuals containing A in both copies (59029-A/A) progress more rapidly to AIDS [54]. Interestingly, the Δ32 phenotype seems to be largely influenced by the presence of mutations in the CCR5 promoter region, with some combinations resulting in poor co-receptor expression and protection against HIV-1 transmission [55].

Antibodies against CCR5 may provide another mechanism of interference with CCR5 function in vivo. Anti-CCR5 antibodies have been reported in a subset of LTNP (24%) but not in other populations studied. These antibodies induce internalization of the co-receptor in vivo and block HIV entry of R5 strains in vitro [56]. Anti-CCR5 antibodies have also been detected in the milk of 66% and 83% of HIV-seronegative and seropositive women, respectively. Anti-CCR5 antibodies purified from these women also protect against infection of R5 strains in vitro [57]. These findings suggest that some degree of protection against vertical transmission of HIV-1 may be mediated by anti-CCR5 antibodies present in the milk. The mechanism governing induction of anti-CCR5 antibodies in humans is unknown, but these observations underscore the interesting prospect of preventing HIV transmission with vaccines targeting the CCR5 co-receptor, an approach that is being examined in murine models [58].

The compelling evidence supporting the role of CCR5Δ32 in protection from disease in homozygous individuals, the apparently healthy characteristics of these individuals, and the ubiquitous presence of CCR5-tropic HIV-1 strains throughout most of the disease, have prompted efforts to target CCR5 with novel antiretroviral therapies. To date, at least nine small-molecule inhibitors and monoclonal antibodies are under development, being tested in clinical trials, or awaiting imminent FDA review [59,60]. Aplaviroc, maraviroc, and vivriviroc are noncompetitive, allosteric CCR5 antagonists that have been tested in large clinical trials. Unlike CCR5 agonists (e.g. PSC-RANTES), none of these compounds induces signaling through CCR5 or receptor internalization. There are potential problems associated with the treatment with CCR5 antagonists. For example, use of this therapy may lead to the emergence of CXCR4-tropic strains, which could accelerate disease progression [61]. In addition, CCR5 inhibition could interfere with the normal immune and inflammatory responses. Despite the apparent normal phenotype of CCR5Δ32 homozygotic individuals, it is not clear how interference with CCR5 will affect the already impaired immune systems in HIV-infected patients. Thus, many questions need to be answered before CCR5 inhibitors are safely used in humans.

### CCR2 and CX3CR1

Despite the pivotal role of CCR5 as the major co-receptor for HIV-1, polymorphisms in other chemokine receptors appear to also exert a certain degree of protection against HIV-1 infection and/or disease progression. The most compelling evidence comes from the CCR2-64I variant, an allelic variant in which isoleucine 64 is replaced by valine [62]. Heterozygous individuals for CCR2-64I progress slower to AIDS, although no clear effect in protecting against HIV-1 infection has been documented. Not all studies have confirmed this association [43,63], and the effect of the CCR2-64I remains controversial. Intriguingly, the CCR2 receptor is used only by a few strains in vivo, thus the mechanism of action of the CCR2-64I variant is unknown. CCR2 lies 17.5 kb upstream of the CCR5 promoter, and it has been suggested that the 64I variant may be in linkage disequilibrium with genetic variations in the CCR5 region [64]. To date, most reports have observed no changes in the levels of CCR2 or CCR5 surface expression in CCR2-64I individuals [64,65]. One study, however, reported lower levels of surface CCR5 and suggested, though it did not prove, that CCR2-64I may bind with increased affinity to CCR5 intracellularly and thus interfere with the expression of CCR5 at the cell surface [66]. Another report suggested interference with CXCR4 as an alternative explanation, demonstrating that the 64I gene product dimerizes with CXCR4 more efficiently than the wild-type CCR2 [67].

CX3CR1, the receptor for the chemokine fractalkine [68], has also been associated with HIV-1 disease progression. Its role as a co-receptor for HIV-1 in vivo is not clear, but it has been suggested that CX3CR1 could affect HIV-1 replication by influencing the recruitment of immunomodulatory cells. Two SNPs have been identified that form part of the haplotype I249-M280. Originally, this haplotype was found at higher frequency in a cohort of Caucasian HIV-infected patients progressing to AIDS faster than normal [69]. These results were later confirmed with a cohort of HIV-infected children [70]. In another study evaluating individuals entering HAART treatment during one year, the I249 polymorphism was found at a higher frequency among those displaying early immunological failure, estimated as a decline in CD4 counts [71]. A study with a Spanish cohort found the haplotype composed of I249 and T280 was overrepresented among LTNPs, as compared to normal progressors, but the same study reported no significant effect on the distribution of the M280 SNP [72]. Adding controversy to the role of CX3CR1, the results with the M280 SNP have not been confirmed in other studies [73-75]. A possible explanation has been proposed to explain these discrepancies, as follows. Due to the deleterious effect of the M280 allele, this SNP may have disappeared from some cohorts due to premature death of patients before recruitment. The low frequency of the allele observed in a French cohort supports this explanation [76]. Additional studies will be needed to address the importance of CX3CR1 in HIV-1 pathogenesis and to understand the biological basis behind the observed phenotypes.

### The phenotypic switch: Does co-receptor usage influence disease progression?

The HIV-1 strains that are most often responsible for transmission utilize CCR5. These so-called M-tropic (R5) viruses predominate during the asymptomatic stage and infect CD4-positive lymphocytes and macrophages. In approximately half of the patients with advanced disease, the viral quasiespecies are dominated by viruses that utilize CXCR4 [77]. These viruses are called T-tropic (or X4) and infect macrophages inefficiently [78,79]. The emergence of X4 viruses ("phenotypic switch") is associated with accelerated decline in CD4-positive lymphocyte counts and faster progression to disease. Thus, the phenotypic switch has been thought of as a causal factor leading to accelerated disease. In support of a role for X4 viruses in disease progression, studies with macaques infected with SIV carrying CXCR4-tropic HIV-1 envelope (SHIV chimera) display rapid loss of CD4 counts and develop AIDS faster than the R5 counterpart [80].

It is possible that the emergence of X4 viruses may be the consequence, rather than the cause of immune deterioration and disease progression [77]. Supporting this idea, not all patients who develop full-blown AIDS experience the switch to X4 viruses, and yet the R5 strains present in these individuals late during disease are more pathogenic than early viruses [81]. The mechanisms governing HIV co-receptor switch are poorly understood. Furthermore, it is not clear whether the so called "switch", emergence of X4 tropic viruses, occurs by acquisition of mutations in R5 envelopes, or rather X4 viruses are transmitted during infection but replicates poorly during the asymptomatic stages. The emergence of viruses with dual tropism (R5-X4) suggest the accumulation of gradual changes. More sensitive phenotypic assays able to detect very small fractions of X4 and dual-tropic viruses will allow us to understand how viral tropism changes throughout infection. Several selective forces have been suggested to explain why the emergence of X4 strains is restricted during initial phases of infection (reviewed in [82]). High levels of CCR5-positive activated and memory cells present in gut-associated lymphoid tissue, an important site during acute infection, may provide fertile ground for the proliferation of R5 tropic strains. In addition, the constitutive levels of expression of SDF-1 in mucosal tissues could act to restrict transmission of X4 viruses [83]. This, however, contradicts the observation that parenteral transmission of HIV-1 also results in the early predominance of R5 viruses. Clearly, some selective forces must keep X4 viruses under control at early stages of infection. Later in infection, the selective pressure achieved by increased levels of β-chemokines (active against R5 strains), and the reduced levels of neutralizing antibodies to which X4 tropic viruses are more sensitive [84] may trigger the phenotypic switch. The observation that many pathogens are potent inducers of HIV-suppressive β-chemokines suggests that opportunistic infections could contribute to the appearance of X4 strains during the symptomatic stage [85].

### HIV-suppressive β-chemokines

The beta-chemokines MIP-1α(CCL3), MIP-1β(CCL4), and RANTES (CCL5) are the natural ligands of CCR5. Two additional variants named CCL3L1 and CCL4L1, encoded by genes arising from the duplication of CCL3 and CCL4, respectively, have also been described [86]. The role of β-chemokines in HIV infection was first proposed in a seminal article in which the anti-HIV-1 effect of these molecules was reported just a few months before the discovery of CCR5 as co-receptor for HIV-1 [87]. Soon thereafter, several reports found inverse correlations between the levels of β-chemokines in plasma and the rate of disease progression [88,89]. Elevated levels of RANTES have also been associated with protection against HIV transmission in some EU cohorts [90]. Interestingly, no differences in the plasma levels of beta-chemokines have been observed in some EU cohorts. These individuals display normal levels of CCR5 on their CD4-positive T cells. However, CD4-positive T cells from these EUs appear more sensitive to the HIV-1 inhibitory effect of β-chemokines, suggesting the existence of yet unknown mechanisms influencing the role of CCR5 in infection [91].

When bound to CCR5, β-chemokines induce internalization of the receptor, abrogating its ability to promote HIV-1 infection [92]. The mechanisms governing inter-individual variations in β-chemokine expression are not completely understood. In support of a role for β-chemokines, SNPs in the MIP-1α gene are found at elevated frequencies among EU individuals [93]. SNPs in the promoter regions of the *RANTES *gene have also been described and they could affect expression of RANTES. However, their role in disease progression remains controversial [94].

CCL3L1, also known as MIP-1αP, is the more potent CCR5 agonist and the strongest inhibitor of infection by R5 HIV-1 strains [95]. Interestingly, the levels of CCL3L1 are determined, in part, by the number of tandem copies of the CCL3L1 gene, which varies from 2–10 among individuals, with highest copy numbers found in African populations [96]. When analyzed by itself, the number of CCL3L1 copies is not significantly associated with HIV susceptibility. However, significant trends are found when the number of copies is analyzed in the context of a specific population. Thus, people with higher number of copies than their ethnic background average are less susceptible to HIV-1 infection and progress slower to AIDS [96]. These findings underscore the important role of CCL3L1 in disease progression.

Another study has revealed a role for MIP-1β in HIV-1 pathogenesis [97]. This chemokine is encoded by two highly related genes (*CCL4 *and *CCL4L1*). Two polymorphisms of the *CCL4L1 *gene have been described (L1 and L2). Individuals homozygous for L2 display reduced levels of CCL4 transcripts when compared to homozygous for L1. A higher frequency of the L2 allele has been observed in a Spanish cohort of HIV-infected patients, as compared to healthy controls, suggesting that the levels of MIP-1β may influence HIV-1 susceptibility [97]. Polymorphisms in the RANTES gene have also been reported. Elevated levels of circulating RANTES are common in EU individuals and in HIV-seropositive patients displaying slow progression (reviewed in [98,99]). Two changes in the promoter (-403G/A and -28C/G) appear to modulate in vitro transcription of RANTES. In vivo, the presence of the -403A and -28G haplotype has been associated with slower disease progression in Japanese and Thai cohorts [100,101], and lower susceptibility to infection in a Chinese cohort [102]. A second study has analyzed only the -403A allele, confirming its protective role in HIV progression, but also describing it as a risk factor for HIV transmission [103]. Thus, the role of polymorphisms in the RANTES promoter remains controversial, with at least two other reports failing to confirm these associations in Spanish cohorts [94,104]. The reason for these discrepancies may be due to the quite different distribution frequencies of RANTES alleles across ethnic groups. Further complicating the study of RANTES polymorphisms, some alleles appear to mitigate the effect of others. Thus, the -28G allele, common in Asians, rare in Euopean-Americans (EA) and absent in Africans, mitigates the disease-accelerating effects of another RANTES variant, In1.1.C, which have been described in EAs [105]. This latter allelic variant by itself potently down-regulates RANTES transcription [106].

SDF-1 (also known as CXCL12) is the only known ligand of CXCR4 [107,108]. As with β-chemokines and CCR5, occupation of CXCR4 by SDF-1 induces internalization of the receptor [109]. Both CXCR4 and SDF-1 are essential during development, and knock out of either of these genes leads to lethal phenotypes in mice [110,111]. Not surprisingly, alleles leading to lack of expression of SDF-1 or CXCR4 have not been identified. Nevertheless, the role of of SDF-1 and CXCR4 in the adult life, recirculating leukocytes and hematopoietic precursors, may be less vital. A polymorphism in the noncoding region of SDF-1 has been reported (SDF1-3'A). In the homozygous form, the presence of an A at position 801 has been associated with slower progression to AIDS, as compared to heterozygous or wild-type homozygous. The biological basis for this association is not clear, since no differences in SDF-1 levels have been observed [112,113]. A number of reports have failed to confirm this association in other cohorts [114-117]. Thus, it is not clear whether the SDF1-3'A variant play a role disease progression.

Other chemokines such as MCP1 (CCL2), MCP3 (CCL7), and eotaxin (CCL11) bind CCR2 and CCR3, but not CCR5. Each of these chemokines have been associated with HIV pathogenesis. It has been suggested that they control migration of immune cells to sites of HIV-1 infection, thus contributing to virus propagation in vivo [118]. Table 1 summarizes the role of known variants of chemokine receptors and their ligands in HIV pathogenesis.

**Table 1:** Chemokine and chemokine receptor variants modulating HIV transmission and pathogenesis

**Gene**	**Allele or factor**	**Mode**	**Effect**	**Mechanism of action**	**Frequency^(1)^**	**References**
**Chemokine Receptor**						

CCR5	Δ32	Recessive	Resistance to infection	Truncated co-receptor is not expressed at the cell surface.	Caucasians (4–15%)	25, 30, 31
CCR5	Δ32	Dominant	Delay AIDS	Reduced co-receptor expression.	Caucasians (4–15%)	38–41
CCR5	C20S	Dominant	Prevent HIV infection in the presence of Δ32	Very low co-receptor expression. Loss of disulfide bridge, improper folding?	Caucasians (0.3%)	49, 51
CCR5	A29S	Unknown ^(2)^	Not evaluated	Failure to bind RANTES, MIP-1β and MIP-1α	Africans (1.5%)	49,51
CCR5	R60S	Unknown ^(2)^	Not evaluated	Poor co-receptor internalization	Africans (1.3%)	51
CCR5	C101X	Dominant	Prevent HIV infection in the presence of Δ32	Truncated co-receptor not expressed at cell surface	Africans (1.4%)	49, 51, 52
CCR5	G106R	Unknown^(2)^	HIV resistance/Delay AIDS?	Very low co-receptor expression	Asians (1.4%)	50
CCR5	C178R	Unknown^(2)^	HIV resistance/Delay AIDS?	Very low co-receptor expression	Asians (0.5%)	49
CCR5	C269F	Unknown^(2)^	HIV resistance/Delay AIDS?	Very low co-receptor expression. Loss of disulfide bridge, improper folding?	Asians (1.4%)	49, 50
CCR5	FS299	Unknown^(2)^	No effect on HIV transmission	Truncated co-receptor, poorly expressed	Asians (4%)	49
CCR5	P1 (promoter haplotype)	Recessive	Accelerate AIDS	Increase CCR5 expression?	Unknown	53
CCR5	59029-A/A (promoter)	Recessive	Accelerate AIDS	Increase CCR5 expression	Caucasians (57%)	54
CCR2	64I	Dominant	Delay AIDS in some cohorts	Influence CCR5 or CXCR4 expression?	General (10–20%)	62, 64, 66, 67
CX3CR1	I249/M280	Recessive	Accelerate AIDS?	Influence recruitment of immune cells?	Caucasians (I249: 26%; M280: 14%)	69, 71

**Chemokine**						

MIP-1αP (CCL3L1)	Gene copy number		Increase susceptibility to infection	Copy number correlates with levels of CCR5 agonist. Block HIV entry	Africans (5–7 mean copy number)	96
MIP-1β(CCL4L1)	L2	Dominant	Increase susceptibility to infection	Reduced level of MIP-1β	Caucasians (16%)	97
RANTES (CCL5)	-403A (promoter)	Dominant	Delay AIDS	Up-regulate RANTES transcription	Asians (27%)	100–102
RANTES (CCL5)	-28G (promoter)	Dominant	Delay AIDS	Up-regulate RANTES transcription	Asians (8%), rare in Caucasians	100–102
RANTES (CCL5)	In1.1C (intronic)	Dominant	Accelerate AIDS	Down-regulate RANTES transcription	General (14–17%)	106
SDF-1 (CXCL12)	3'A	Recessive	Delay AIDS?	Unknown.	Asians (25–35%) Oceanian (50–70%)	112, 113
MCP1/MCP3/Eotaxin	H7 haplotype	Dominant	Decrease susceptibility to infection	Unknown immunomodulatory effects	Caucasians (19%)	118

^(1) ^Allele frequency in populations in which the variant is more predominant. ^(2) ^No homozygous individuals have been identified

### DC-SIGN

DC-SIGN is a mannose-binding, calcium-dependent lectin that has been involved in transmission of HIV-1 from dendritic cells (DC) to T lymphocytes, a phenomenom named "trans-enhancement" (reviewed in [119,120]). DC-SIGN is expressed on immature DCs and activated B lymphocytes. Trans-enhancement requires binding of HIV-1 particles to DC-SIGN via the high mannose glycans present in gp120. The mechanism of transfer of HIV-1 to T cells remains controversial. First, it was proposed that transfer requires internalization and transient storage of HIV-1 particles in subcellular compartments [121]. Recent evidence suggests that infection of DC cells is required for efficient transfer of HIV-1 to other cells [122].

DCs are thought to be among the first cells infected by HIV on the genital mucosa. Infected DCs migrate to lymph nodes where they transfer viruses to T cells. By infecting the very same cells implicated in protection against infection in mucosal tissue, HIV-1 utilizes DCs as Trojan horses that spread the virus to the lymph nodes. The role played by DCs in facilitating infection suggests a possible role for DC-SIGN variants and other C-type lectins in HIV-1 disease progression and transmission. A polymorphism in the DC-SIGN promoter at positions -336 has been identified. Individuals at risk of HIV carrying the -336C allele are more susceptible to infection than persons with the -336T variant [123]. This association, however, has been observed for parenteral transmission of HIV-1, but not for mucosally acquired infection. Variants in the coding region of DC-SIGN and DC-SIGNR have also been identified. However, the importance of these alleles in protecting from HIV-1 infection has yet not been fully elucidated [124-126].

Langerin, also called CD207, is selectively expressed in Langerhans cells, which are spread over the mucosa through which HIV transmission occurs. Under some experimental conditions, Langerhans cells are infected with HIV-1 and transmit virions to T cells [127]. However, recent evidence suggests that Langerin, in contrast to DC-SIGN, prevents HIV-1 transmission. HIV-1 particles captured by Langerin are internalized and degraded into Birbeck granules [128]. Thus, Langerhans cells appear to present a first barrier against infection. This study does not exclude the possibility that these cells transmit HIV-1 at high viral inocula [129]. The role of Langerin variants in HIV-1 transmission has not been studied, although a mutation in the *langerin *gene in a person deficient in Birbeck granules has been described [130].

In addition to DC SIGN, other C-type lectin receptors may also act as receptors for HIV-1: DC-SIGN-related (DC-SIGNR), the mannose receptor (MR), and Langerin can also bind gp120 [120].

### Anti-HIV-1 activity of human defensins

Defensins are small antimicrobial and antiviral cysteine-rich cationic peptides produced by leukocytes and epithelial cells. The role of defensins in innate immunity to fight bacterial, viral and fungal infections has long been known [131,132](reviewed in [133-135]). The first report on the anti-HIV-1 activity of defensins dates back to 1993 [136].

Mammalian defensins are classified into alpha-, beta-, and theta defensins, and differ in their size and distribution of disulfide bridges [135]. Alpha and β-defensins are peptides typically composed of 30–45 residues, and both display anti HIV-1 activity [135]. Theta-defensins are cyclic peptides composed of two alpha-like precursor peptides. Active theta-defensin products are found only in some non-human primates, and are typically composed of 16–18 residues [137]. Given their smaller size theta-defensins have been included in the family of minidefensins, which include molecules also found in other species such as horseshoe crabs and spiders [138]. In humans and chimpanzees theta-defensins are found only as inactive pseudogenes. These pseudogenes are transcribed into mRNAs, but they harbor premature stop codons that preclude expression of functional products. Interestingly, when the putative human ancestral gene for a human theta-defensin was reconstituted, it was found to display in vitro potent anti-HIV-1 activity against R5 and X4 strains [139]. The artificially reconstituted product was named human retrocyclin-1. This molecule displays lectin-like properties and binds to CD4 and gp120, thus preventing entry of HIV-1 into target cells [140].

So far six human α-defensins (also known as human neutrophil peptides or HNP) have been identified. α-defensins also bind CD4 and the viral envelope glycoprotein. Treatment of permissive cells with α-defensins induces down-modulation of CD4 [141]. Additionally, in the absence of serum (e.g. at mucosal surfaces), α-defensins may inactivate virion particles by a mechanism that includes membrane disruption [142]. These findings indicate that α-defensins block HIV-1 entry at several steps, by directly inactivating virions and by blocking or eliminating the viral receptor from the cell surface. The mechanism of action of α-defensins and their inhibition profile led researchers to suggest that these molecules could constitute the long-sought CD8-positive cell anti-HIV factor (CAF) [143]. The existence of CAF was first suggested in 1986, as a soluble factor derived from CD8-positive cells in LTNP individuals with the ability to achieve durable immune responses controlling HIV-1 infection [144]. A report in 2002 suggested that based on specific antibody recognition, α-defensins 1, 2, and 3 were responsible for the antiviral activity of the eluded CAF factor. Experiments demonstrated that CAF activity could be eliminated with anti-α-defensins antibodies, and α-defensins could be detected inside CD8-positive cells [143]. Later reports failed to confirm these results and found no evidence for the production of α-defensins in CD8-positive cells, although CD8-positive cells may internalize α-defensins. However, it is not clear whether the uptake of these molecules is needed for their function [145,146]. Beta-chemokines produced by CD8-positive cells may also contribute to the activity of CAF, which may no longer be ascribed to a single host factor. It is likely that CD8-positive cells express an unknown array of novel HIV-suppressive factors. Thus, the search to elucidate the CAF factor activities is still ongoing.

Six human β-defensins have been identified in epithelial cells, although genomic searches indicate that up to 28 different genes may be present in humans [147]. The mechanism of action of β-defensins shares some similarities with that of α-defensins. They block viral entry of both X4- and R5-tropic HIV-1 strains, although their effect is more potent with T-tropic isolates. Beta-defensins 2 and 3 achieve their inhibitory effect in part by direct inactivation of viral particles, and also by down-modulation of CXCR4, but not CCR5, on T cells [148]. This latter mechanism, however, has not been confirmed by others [149]. Interestingly, HIV-1 induces expression of human β-defensins 2 and 3, but not 1, the latter of which displays poor antiviral activity. Induction of these defensins occurs by a mechanism that does not require viral replication [148,150,151]. Recent findings suggest that β-defensin 2 may mediate its effect via CCR6. Beta-defensin 2 binds CCR6, and its inhibitory effect is abrogated after treatment of cells with anti-CCR6 antibodies. In agreement with this, MIP-3-α(also known as CCL20), the cognate ligand for CCR6, blocks HIV-1 infection in a manner similar to β-defensin 2 [152]. CCR6 is not a co-receptor for HIV-1, but is expressed in CD4+CD45RO+CCR5+ lymphocytes and dendritic cells, where it may play an important role governing the movement of immune cells to mucosal surfaces, the first tissues encountered by HIV during sexual transmission. Thus, β-defensins may also control HIV-1 replication by modulating the immune system. The mechanisms of defense against HIV infection mediated by α-, β-, and θ-defensins are summarized in Table 2.

**Table 2:** Anti-HIV activity of human defensins

**Defensins**	**Regulation**	**Cell Source**	**Mechanisms**	**References**
**α-Defensins**				

HNP1, HNP2, and HNP3	Constitutive HPN2 may be the product of proteolytic processing of HNP1/HPN3	Neutrophils and promyelocytes	• CD4 down-modulation • Viral membrane disruption and binding to CD4 and gp120 (in absence of serum) • Upregulation of CC-chemokines in macrophages • Block of nuclear transport (by HNP1)	131, 143, 146
HNP4	Constitutive	Neutrophils	• Unknown (lectin-independent mechanism)	135

**β-Defensins**				

HBD2 and HBD3	Inducible by HIV, opportunistic infections, and pro-inflammatory cytokines (TNF, IL-1B)	Epithelial cells, monocytes, monocytes-derived DCs, macrophages, and keratinocytes	• Viral membrane disruption (absence of serum) • CXCR4 down-modulation • CCR6-mediated chemotactic effects	148–151

**θ-Defensins**				

Retrocyclins (RTD1, RTD2)	Synthesis blocked in humans by premature termination codon	RNA transcripts, not protein, expressed in bone marrow	• Prevent HIV entry by binding to CD4 and gp120	138–140

The demonstrated antiviral activity of defensins have encouraged the search for polymorphisms influencing HIV-1 infection and disease progression. Both, α-, and β-defensins have been found in human breast milk, suggesting that they could play a role in protecting infants from infection [153,154]. One study has associated the amount of α-defensins in breast milk with protection against intrapartum and postnatal transmission of HIV-1 [155]. SNPs in the 5'-untranslated region of the human β-defensin 1 gene (DEFB1) have been associated with HIV-1 infection in an Italian pediatric cohort. The presence of a C/C allele at position -44 in HIV-1 infected mothers and their children is associated with higher risk of maternal-fetal transmission [156,157]. These results have been confirmed by the same group in another cohort of Brazilian children [158]. However, studies with HIV-1 infected adults are still missing. Further highlighting the role of human defensins in HIV pathogenesis, elevated levels of α-defensins have been reported in EU individuals and HIV-infected women, as compared to healthy controls [159]. Interestingly, human genes for both α-defensins (DEFA1 and DEFA3), and β-defensins (DEFB4, DEFB103, and DEFB104) are known to be polymorphic in copy number [160-162]. Transcription of these genes correlates with copy number, and it is tempting to speculate that, as demonstrated for the CCL3L1 and CCL4L1 chemokines, polymorphisms in the copy number of defensin genes may modulate HIV-1 susceptibility and disease progression.

## Host factors modulating early post-entry events of HIV-1 replication

### Cyclophilin A and TRIM5α

Studies in the early '80s identified cyclophilin A (CypA) as a cytosolic protein binding to the immunosuppressant molecule cyclosporin A [163]. Subsequent studies identified an anti-HIV activity of cyclosporin A [164,165], although its mechanism of action was not elucidated at that time. The role of CypA in HIV-1 replication was investigated in detail after discovering its HIV-1 capsid (CA) binding properties in a yeast two-hybrid screen [166]. As a result of this interaction CypA is incorporated into virion particles [167-169]. After years of studies the mechanism of action of CypA has just begun to be unraveled. HIV-1 has a limited host range that appears to be explained in part by the existence of saturable inhibitory factors that block virus replication at early steps, before reverse transcription occurs [170-172]. CypA promotes HIV-1 infectivity in target cells by a mechanism that does not require CypA incorporation into virions [173,174]. The exact mechanism of action remains an enigma. CypA appears to modulate the action of other restriction factor(s), perhaps by altering CA conformation in a manner that makes it less sensitive to their inhibitory effect (reviewed in [175]). The nature of the proposed conformational change may relate to the cis-trans isomerization of peptidyl-prolyl bonds mediated by CypA. However, the significance of this activity has not been conclusively demonstrated [176]. Interestingly, cyclophilins may constitute modulators of the innate immune response in other eukaryotic systems. Recently, an innate mechanism of defense against *Pseudomonas syringae *infection has been described in *Arabidopsis*. Upon infection, a plant cyclophilin activates a bacterial protease, which then triggers a cascade of events that activates a plant's defense response [177]. Thus, cyclophilins may represent evolutionarily conserved mechanisms of innate defense.

Several SNPs have been identified in the human CypA gene (*PP1A*). Their associations with HIV-1 infection and disease progression in vivo have not been studied to date. However, one group has used an ex vivo approach to evaluate polymorphisms in *PP1A*. In this strategy, CD4 T cells were purified from healthy donors and challenged in vitro with HIV-1. The extent of viral replication was correlated with specific alleles, and when in vivo data is available, correlations with disease progression in a cohort of HIV-1 infected individuals were sought. Following this approach a polymorphism in the PP1A promoter (1650A/G) was associated with lower ex vivo virus replication in cells derived from PP1A homozygous individuals (AA), and slower disease progression in vivo [12]. The ex vivo replication profile in cells carrying alleles previously associated with slow progression (CCR5Δ32 and CCR264I) or rapid disease progression (RANTES In1.1C) followed the expected trend, confirming the validity of this ex vivo approach to identify relevant alleles. Nevertheless, validation studies with other cohorts will be needed to define the role of CypA variants in HIV-1 pathogenesis.

Another cellular factor implicated in early steps of HIV-1 replication is TRIM5α. This protein was identified from a rhesus macaque library screened for simian factors restricting HIV-1 replication upon transfer into otherwise permissive human cells [178]. Early studies suggested that the action of TRIM5α and CypA were somehow related, and as mentioned above, it was suggested that CypA protects CA from the deleterious effects of human TRIM5α [175]. TRIM5α also binds HIV-1 CA, though apparently in a CypA-independent manner [179-181]. An alternative model proposes that common restriction factors may be shared in the cascade of events that leads to inhibition of HIV-1 replication by TRIM5α, and promotion of infectivity by CypA [175].

Unlike simian forms, the human ortholog of TRIM5α appears to only modestly inhibit HIV-1 replication. This has led to the hypothesis that polymorphisms in the human TRIM5α gene might result in increased restriction and modulate HIV-1 infection. One recent report has identified a TRIM5α haplotype (hap 9) containing a nonsynonymous SNP at position 136 (R136Q) that occurs with higher frequency among HIV-infected subjects than in exposed seronegative individuals [182]. None of the individual SNPs found in this haplotype influenced HIV transmission, suggesting that the genetic sequence responsible for the observed phenotype is in linkage disequilibrium with this haplotype, lying in *TRIM5 *or one of the *TRIM *genes adjacent to it. Another study evaluated the 136Q allele and found the frequency of this allele elevated in uninfected African-Americans, as compared to HIV-1 infected subjects. However, this correlation was not observed in individuals of European descent. In agreement with a role for TRIM5α, the 136Q variant exhibited slightly stronger inhibitory activity than the 136R counterpart [183]. The same study reported the identification of two additional non-coding SNPs present in one TRIM5α haplotype that is found in HIV-1 infected patients at higher frequencies than in uninfected individuals. The above studies suggest that polymorphisms in TRIM5α may influence susceptibility to HIV-1 infection. Only one study has evaluated the role of common TRIM5α variants on disease progression. The authors analyzed a cohort of 979 HIV-infected patients and found only modest associations with the rate of CD4-positive T cell decline before the onset of treatment [184]. The viral determinants for sensitivity to inhibition by TRIM5α lie in the viral CA region. In vitro, mutations in HIV-1 CA overcome the restriction of HIV-1 to replicate in simian cells. A study by the Schuitemaker group has evaluated the presence of TRIM5α escape mutants in HIV-1 infected individuals, as an indicator of TRIM5α-mediated inhibition. Interestingly, CA escape mutants emerging late in the infection process could be found in 14% of the infected patients (N.A. Kootstra and H. Shuitemaker, personal communication). These findings suggest the existence of selective pressure on the virus to avoid TRIM5α-mediated restriction.

### The innate immune response mediated by APOBEC3G

Host cells are endowed with another mechanism to halt HIV-1 infection before integration occurs. The human apolipoprotein B mRNA-editing enzyme-catalytic polypeptide-like-3G (APOBEC3G), formerly known as CEM15, is an endogenous inhibitor of HIV-1 replication [185-187]. In the absence of the viral protein Vif, APOBEC3G is incorporated into HIV-1 particles in the producer cell, and during reverse transcription deaminates cytosine bases to uracil in the negative-sense single-stranded DNA, resulting in G to A hypermutations in the complementary, positive sense DNA strand. This hypermutation leaves the viral cDNA vulnerable to degradation by nucleases. Those cDNAs that manage to integrate into the host chromosomes carry multiple mutations that likely result in aberrant viral products [188-190]. Recent reports appear to suggest that APOBEC3G also exerts antiviral activities through mechanisms independent of its cytidine deaminase activity [191,192]. APOBEC3G can be found in cells in two different forms of low and high molecular-mass (LMM and HMM, respectively). APOBEC3G in LMM form is enzymatically active and restricts HIV-1 infection. The HMM form is a catalytically inactive ribonucleoprotein complex that appears to protect against mobilization of endogenous cellular retroelements such as Alu and hY [193-195]. Endogenous retroelements can influence transcription of adjacent genes and induce genetic disease in humans by insertional inactivation [196-198]. This APOBEC3G-induced mechanism of restriction of Alu and hY mobilization is mediated by sequestration of the RNA retroelements into HMM complexes, which are kept away from the L1-dependent retrotransposition machinery [193]. APOBEC3G and related deaminases may also protect against other retroviruses and hepatitis B virus, which also relies on a reverse transcription step to complete its life cycle [186,191].

The Vif protein binds APOBEC3G in virus producer cells and targets it for degradation in the proteasome, thus preventing APOBEC3G incorporation into virions [199-201]. To induce APOBEC3G degradation Vif binds the cellular proteins Cul5, elonginB, elonginC and Rbx1, to form a cullin5-based E3 ubiquitin ligase complex that leads to polyubiquitination and ultimately to proteasomal degradation of APOBEC3G and APOBEC3F, which also displays anti-HIV activity [200,202].

The importance of the APOBEC3G-mediated innate response against HIV-1 is underscored by the existence of both APOBEC3G and Vif genetic variants influencing disease progression. Studies with LTNP have revealed an association between low viral load and a serine residue at position 132 of Vif. When tested in vitro, HIV-1 carrying Ser132 displayed a five-fold decrease in replication in PBMC, as compared to virus containing the wild type allele (Arg) at the same position [203]. Another study revealed the presence of a two-amino-acid insertion in the Vif protein of a mother's virus and her child's, both LTNP. This amino acid insertion results in an aberrant Vif protein that severely impairs replication when expressed in a recombinant HIV-1 [204]. Furthermore, Vif-defective proteins have been isolated from strains from other LTNP individuals [205]. The reason why some of these LTNP Vif variants do not revert to encode a functional product is not understood. It is possible that Vif plays other roles in viral replication that constrain its ability to mutate. Alternatively, the genetic background of these LTNPs may have weakened the virus' ability to replicate and mutate in these subjects.

With regards to APOBEC3G, it is plausible that genetic variants with altered inhibitory activity or partially resistant to Vif might alter the course of HIV-1 disease. A variant of APOBEC3G common in African-Americans has been identified, which contains a non-synonymous substitution. This allele carries Arg instead of His at position 186 (186R) and is strongly associated with faster decline of CD4 T cells and accelerated progression to AIDS [206]. This variant, however, appears to have the same in vitro inhibitory activity than the common 186H allele. An extensive analysis of a French cohort failed to identify significant associations between 29 APOBEC3G polymorphisms and disease progression, although discrepancies with previous studies may be explained by the different variables used to analyze disease progression [207]. Another study identified an APOBEC3G variant (C40693T) in a cohort of EU Caucasians subjects. This allele was found to be strongly associated with increased risk of infection [208]. A recent report revealed an inverse correlation between APOBEC3G mRNA levels and viral load in infected individuals, with the highest APOBEC3G levels observed in LTNP patients [209]. However, no associations with specific variants in the APOBEC3G gene have been identified in these individuals. The controversy over APOBEC3G variants, and the lack of a potential mechanism to explain their phenotypes warrants the need for additional studies.

A recent study has evaluated polymorphisms in the *CUL5 *gene encoding the Cullin 5 protein. Haplotypes encompassing 12 SNPs were clustered into two different groups that segregated with different profiles of CD4 T cell decline rates and viral load [210]. One individual SNP (SNP6 A/G) appears responsible for the rapid CD4 T cell depletion and progression to AIDS. This correlation was observed in African-Americans, but not found in European-American and Asian populations. Interestingly, a significant additive effect was observed between some of the SNPs found in *CUL5 *and the APOBEC3G-186H allele. This study highlights the role of Cullin 5 in HIV-1 pathogenesis. Additional studies will be needed to understand the roles of *CUL5 *variants. Table 3 summarizes the effect on HIV infection and progression to AIDS of known genetic variants of human genes participating in post-entry steps of the virus life cycle.

**Table 3:** Human genes modulating HIV pathogenesis by influencing post-entry steps of the viral life cycle

**Gene**	**Allele or factor**	**Mode**	**Effect**	**Mechanism of action**	**Frequency^(1)^**	**References**
CypA (PP1A)	1650 G	Recessive	Delay AIDS	Unknown	Unknown	12
TRIM5α	Haplotype 9		Increase HIV transmission	Unknown	Caucasians (1%)	182
TRIM5α	136Q	Dominant	Protect against HIV infection	136Q variant displays stronger anti-HIV activity	African Americans (20%)	183
TRIM5α	43Y	Dominant	Protect against HIV infection	Unknown	African Americans (6.5%)	183
APOBEC3G	186R	Recessive	Accelerate AIDS	Unknown	African Americans (36.7%)	206
APOBEC3G	C40693T	Unknown	Increase HIV transmission	Unknown (variant found in intronic region)	Caucasians (< 1%)	208
Cullin5	SNP6 A/G	Dominant (additive)	Accelerated CD4 T cell depletion and AIDS progression	Unknown (the SNP6 G product displays stronger binding to nuclear proteins	Africans (5%)^(2)^	210
TSG101	-183C	Dominant	Accelerated CD4 T cell decline	Increase virus budding? (paradoxically the -183C variant reduces replication in ex-vivo systems)	Caucasians (17%)	12, 229

^(1) ^Allele frequency in populations in which the variant is more predominant. ^(2) ^Higher allele frequencies are observed in European Americans (10%) and Chinese (20%), however the correlation is not observed in these populations.

## Cellular factors modulating late steps of HIV-1 replication

The final steps of HIV-1 replication involve viral assembly and the release of new progeny. HIV-1 encodes the viral protein U (Vpu), which facilitates viral release from infected cells. Vpu appears to overcome the inhibitory effect of cellular factor(s) blocking viral budding [211,212]. The identity of this factor remains elusive. Recent evidence suggests that this factor may redirect HIV-1 particles to an endocytic route, a phenomenon that is overcome by expression of Vpu [212]. Some studies have suggested that Vpu may act by counteracting the potassium channel TASK-1, which interferes with the release of retroviruses by unknown mechanisms [213,214]. Vpu is also known to interact with βTRCP, a component of a cullin1-based E3 ubiquitin ligase. Interaction between Vpu and βTRCP targets CD4 for proteasomal degradation, contributing to virus-induced down-modulation of CD4. By binding to βTRCP, Vpu also inhibits the βTRCP-dependent degradation of IκB, which results in suppression of NF-κB and induction of apoptosis in infected cells [215,216]. Therefore, it is plausible that the levels of these proteins or polymorphisms in cellular genes implicated in Vpu function modulate HIV-1 susceptibility and disease progression in vivo.

The cellular determinants responsible for assembly and release of HIV-1 have been extensively documented in the last five years. TSG101 (tumor susceptibility gene 101) encodes a host cellular protein required for HIV-1 budding. The PTAP "late domain" found in the P6 product of HIV-1 Gag recruits TSG101 (also known as VPS23) to facilitate virus budding [217-219]. In uninfected cells TSG101 functions in the biogenesis of the multivesicular body (MVB), which is required for the sorting of mono-ubiquitinated transmembrane proteins into internal vesicles. HIV-1 P6, through its interaction with TSG101, usurps the cellular machinery to promote viral budding at the plasma membrane in T cells and macrophages [220-222](reviewed in [223,224]). TSG101 is a component of the ESCRT-1 complex (endosomal sorting complex required for transport), and is known to bind multiple components of the MVB machinery (VPS28, VPS37, AIP1, Hrs, TOM1L1, EAP30, EAP45, and GGA proteins). At least eleven different human proteins have been shown to modulate HIV-1 budding (TSG101, VPS28, VPS37C, CHMP2A, CHMP3, CHMP4B, CHMP4C, VPS4A, VPS4B, AIP1, and Tal), and it is likely that many other components of the MVB pathway participate in this step of the viral cycle [223-228].

A few studies have evaluated polymorphisms in TSG101. One study utilized an ex-vivo/in vivo approach to evaluate a SNP in the noncoding region of the *tsg101 *gene (position -183). The presence of C at this position (instead of T) results in a dominant effect that significantly reduces replication of HIV-1 in vitro [12]. Paradoxically, the same allele leads to faster disease progression (measured as CD4 cell count decline). Thus the role of this variant in HIV pathogenesis remains unclear [12,229].

It has long been known that interferon-α, a key component of the innate response against some viral infections, interferes with HIV-1 particle release, however its mechanism of action has remained unknown until recently [230,231]. The interferon-induced ubiquitin-like protein ISG15 appears to be a critical component of the interferon-α antiviral response. ISG15 inhibits ubiquitination of TSG101 and the late HIV-1 Gag domain, inhibiting the P6-TSG101 interaction required for viral budding [232].

Another cellular factor regulating late steps of the viral cycle is HP68 (also known as ABCE1 and RNase L inhibitor). HP68 appears to be critical in the assembly of the HIV-1 capsid proteins. HP68 interacts with the NC domain of Gag, and as proteolytic processing of the Gag polypeptide occurs, HP68 is released from these complexes and viral particles acquire their mature conformation [233-235]. The role of HP68 in HIV pathogenesis in vivo has not yet been evaluated.

The matrix region of HIV-1 Gag binds the delta subunit of the adaptor protein complex 3 (AP-3). This interaction appears to play an important role in particle assembly [236]. Although its role in HIV pathogenesis has not been studied, mutations in ADTB3A, which codes for the beta3A subunit of AP-3, have been characterized in individuals with a rare form of Hermansky-Pudlak syndrome [237]. These individuals present defects in the vesicular trafficking pathways leading to formation of the melanosomal compartment, and are characterized by the presence of oculocutaneous albinism.

### Cellular factors incorporated into HIV-1 particles

Many cellular proteins known to play important roles in HIV-1 pathogenesis are incorporated into virions. For instance, CypA, APOBEC3G, TSG101, and VPS28, which are required at different steps of the viral cycle, are encapsidated into virions [167,189,217,238]. Most cellular proteins are incorporated into viral membranes in a passive manner if they associate with areas of the cell membrane through which the virus buds (reviewed in [239]). However, some proteins are specifically incorporated into viral membranes through interaction with viral products. Specific incorporation of cellular proteins inside virions or into viral membranes has generally been seen as suggestive of a functional role in the HIV-1 life cycle. Thus, it is expected that many other proteins known to associate with virions play a role in HIV-1 transmission and disease progression. Among these potential candidates are the integral membrane-spanning intracellular adhesion molecule 1 (ICAM-1). ICAM-1 expression drastically enhances HIV-1 infectivity, and makes virions more resistant to neutralization with gp120-specific antibodies or sera from HIV-infected patients [240-245]. Enhancement of infectivity can be blocked with anti-ICAM-1 mAbs, and is augmented in cells expressing higher levels of LFA-1, the ICAM-1 receptor. These findings suggest that HIV-1 may use an ICAM-1/LFA-1 dependent route to promote cellular entry and escape the humoral immune responses targeting the viral envelope protein. The interaction between ICAM-1 and LFA-1 may also play an important role in infection of CD4-positive memory T cells, which are considered a latent reservoir of the virus [246]. Polymorphisms in the ICAM-1 gene have been associated with susceptibility to a range of degenerative and inflammatory diseases. However their role in HIV pathogenesis has not been evaluated [247].

Specific incorporation into HIV-1 virions of host-derived HLA-DR, β2-microglobulin and class I MHC molecules has long been known [248,249]. However, the physiological relevance of these associations is poorly understood. Several explanations have been suggested. Interestingly, immunization with HLA-DR and class I MHC proteins confer protection against SIV infection, probably by generating class-I and class-II-specific antibodies [250,251]. Additionally, incorporation of HLA-DR, one of the most abundant cellular proteins, into viral membranes, renders virions capable of stimulating purified T lymphocytes in the presence of the staphylococcal enterotoxin A superantigen [252]. Evidence that these mechanisms may influence HIV-1 pathogenesis is still missing, and these studies should be interpreted with caution. It has also been suggested that virion incorporation of MHC molecules could enhance virus infectivity [239].

Other proteins specifically incorporated into lentivirus capsids are hsp70, Ini1/SNF5, and the lysyl-tRNA synthetase. Different roles in the viral life cycle have been ascribed to these proteins. Hsp70 enhances the infectivity of HIV-1 [253], whereas Ini1/SNF5 appears to modulate virus yield and infectivity [254]. Lysyl-tRNA synthetase may participate in the recruitment of tRNALys to prime viral DNA synthesis [255-257]. The role of these proteins in HIV-1 pathogenesis in vivo has not been addressed.

### Host proteins modulating HIV-1 envelope incorporation

There is mounting evidence that the levels of envelope in HIV-1 particles are finely modulated and could play an important role in viral pathogenesis. Recent studies have changed the common view of the HIV-1 virion. Initially, it was believed that, like in other enveloped viruses, HIV-1 viral membranes were abundant in envelope proteins. It is now known that the relative density of envelope spikes in HIV-1 virions is surprisingly low. Compared to influenza, which contains 200–300 spikes per particle, it is estimated that HIV-1 virions incorporate only 4 to 7 spikes per particle [258]. The reason for maintaining envelope levels to a minimum is unknown, though it may be related to the need to evade the antibody neutralizing response directed against the viral envelope.

In HIV-1, specific signal sequences in the cytoplasmic and membrane domain of gp41 modulate expression of envelope at the cell surface. Interactions between the cytoplasmic domain of gp41 and the viral matrix protein modulates envelope incorporation by a mechanism that requires the cellular protein TIP47 [259-264]. The CD4 receptor, which plays an essential role allowing viral entry, inhibits envelope incorporation late in the viral cycle (reviewed in [265]). Excessive amounts of CD4 in virus-producer cells interfere with envelope transport to the cell surface, preventing its incorporation into viral membranes [266,267]. Additionally, CD4, which is normally excluded from viral membranes, is then incorporated into HIV-1, interfering with the ability of envelope to bind CD4 in target cells. To overcome the inhibitory effects of CD4, HIV-1 utilizes three viral products, Nef, Vpu, and Envelope, to ensure CD4 is removed after its function in viral entry has been performed [265,268,269]. The importance of CD4 down-modulation in HIV-1 replication has been highlighted in recent studies. Nef-mediated CD4 down-regulation correlates with the ability of HIV-1 to replicate in blood-derived primary lymphocytes and human lymphoid tissue [270-272]. Other studies have evaluated the Nef-dependent CD4 down-modulation activity through different stages of disease. Nef alleles isolated from late symptomatic stages are more potent CD4 down-modulators than alleles isolated from the chronic asymptomatic stage [273,274]. This enhanced activity translates in faster HIV-1 replicative properties in vitro, and presumably in enhanced pathogenicity in vivo. Many other studies have revealed the existence of mutations or deletions in *nef *genes isolated from LTNP patients. In some instances, these changes have been associated with diminished CD4 down-modulation activity [275-278].

No enzymatic activities have been associated to HIV-1 Nef, and it is widely accepted that this viral product exerts its CD4 down-modulation activity by binding to host proteins. Nef is known to interact with more than 25 cellular proteins. The physiological relevance of these interactions, however, has not always been evaluated [279]. Despite the potential role of cellular partners involved in Nef activities, to date no studies evaluating their influence in HIV-1 pathogenesis have been performed.

### Host factors modulating the innate immune response against HIV-1 infection

#### The cytokine system and its role in immunity against HIV-1

Cytokines play an important role in controlling the homeostasis of the immune system and the response against pathogens. The role of IL-1, IL-2, IL-4, IL-6, IL-10, IL-16 and IL-18 in HIV-1 disease progression has been amply documented, as have IFN- α, IFN-β, TNF-α, and TNF-β.

With regards to HIV infection, cytokines can be grouped into HIV-inducing (TNF- α, TNF-β, IL-1, and IL-6), and HIV-suppressive (IFN-α, IFN-β, and IL-16). IFN-gamma and IL-4 display both stimulatory and anti-HIV effects [280]. IL-1α and IL-1β are co-stimulatory cytokines for T helper cells and promote maturation and clonal expansion of B cells [281]. The role of the IL-1-dependent inflammatory response has been highlighted by the identification of polymorphisms of the IL-1 receptor gene (IL1Rα). The SNP IL1Rα-2134 has been found at higher frequency in a French cohort of slow progressors [282]. IL-2, a potent inducer of proliferation of antigen-primed T lymphocytes, also plays an essential role in immune regulation. Deficiency in IL-2 production is one of the first immunologic defects described in HIV-1 infected individuals [283]. Polymorphisms in the IL-2 gene have been extensively analyzed in a study of HIV-seropositive African-Americans [284]. A SNP deletion in a noncoding region of IL-2 (IL2+3896) was associated with disease progression in homozygotic individuals.

IL-4 is a Th2 cytokine with co-stimulatory activity for T and B cells. It has opposite effects on the HIV-1 co-receptors, upregulating CCR5 and down-modulating CXCR4, although it is not clear whether modulation of the HIV-1 co-receptors is sufficient to influence viral entry. Other IL-4-dependent inhibitory effects in HIV-1 replication have been documented, although these appear to be cell-dependent and mostly affect X4-tropic strains. An IL-4 SNP (589T) known to influence transcription of the cytokine has been associated with progression to AIDS in some but not all studied cohorts [285,286]. Genetic studies of the IL-4 receptor alpha chain gene have also found significant associations with disease progression and susceptibility to HIV-1 infection, although this latter phenotype appears to depend on the route of transmission [287].

IL-16, a ligand for CD4, is a multifunctional cytokine. IL-16 induces expression of class II MHC and displays chemotactic properties on CD4-positive T cells and monocytes. IL-16 binds CD4 at an epitope distinct from from the HIV-1 envelope binding site. The cytokine inhibits HIV-1 transcription in T lymphocytes and monocytic cells [288,289]. Several studies have described the influence of IL-16 in HIV-1 infection. The protective role of IL-16 appears to occur at two different levels. First, IL-16 suppresses HIV-1 replication. Second, IL-16 facilitates immune reconstitution by enhancing expression of IL-2 receptors and making T cells responsive to IL-2 [290]. Interestingly, T cell clones from LTNP individuals express higher levels of IL-16 than cells from AIDS patients [291]. Other clinical studies have found decreased levels of IL-16 as disease progression advances [292,293]. Polymorphisms in the IL-16 gene have been identified, but no associations with HIV/AIDS have been reported to date [294].

IL-18 is a pro-inflammatory cytokine mostly secreted by activated macrophages. IL-18 is known to play an important role in responses against viruses and intracellular pathogens. IL-18 induces IFN-γ production in T cells and enhances NK cytotoxic activity. Increased levels of IL-18 have been found in late stage HIV-1 patients, and plasma levels of this cytokine correlate with viral load, constituting an excellent marker for disease progression [295-297]. Several polymorphisms in the IL-18 gene are known, and at least one SNP (IL18 C607A) has been associated with increased risk of infection in a pediatric cohort [298,299].

Proteins of the interferon family play essential roles in the innate response against viruses, including HIV-1. Type 1 interferons (IFN-α and IFN-β) induce expression of protein kinase R (PKR), which blocks protein synthesis in virus-infected cells. IFN-α/β also enhance lytic activity of NK cells, making them very effective in killing virally-infected cells [300]. Polymorphisms in the IFN-α receptor 1 (IFNAR1) have been associated with disease progression and susceptibility to HIV-1 infection [301].

### Toll-like receptors

Another key family of proteins participating in the innate immune response against pathogens is the Toll-like-receptors (TLR) family. During HIV infection, TLRs may play an important role in modulating the extent of viral replication (reviewed in [302,303]). There is mounting evidence that in addition to direct activation of TLRs by HIV, indirect activation mediated by opportunistic pathogens may influence the outcome of disease [304,305]. Signals mediated by pathogens can activate HIV transcription in trans, via the production of proinflammatory cytokines such as TNF-α, IL-1, or in cis, directly inducing expression of cellular factors activating the viral LTR (NF-κB and AP-1) [302].

Eleven TLR members have been identified so far in humans [306]. TLRs recognize specific motifs expressed by pathogens such as lipid moieties, protein motifs, and nucleotide sequences. The first piece of evidence suggesting a role for TLRs in HIV-1 pathogenesis dates back to 1990, when it was shown that bacterial LPS activates the viral LTR, a process later found to be mediated by TLR4 [307]. To date, at least five TLR members (TLR2, TLR4, TLR7, TLR8, and TLR9) have been implicated in induction of HIV-1 expression during opportunistic co-infections [308-313]. Several studies support a role for co-infecting pathogens in HIV transmission and disease. TNF-α levels are elevated in HIV-infected patients co-infected with *Mycobacterium tuberculosis *[314]. In another report, a clinical trial evaluating antisense molecules targeting HIV-1 Gag produced unexpected results, and revealed elevated viral loads in treated patients. Several clues led the authors to conclude that the unwanted effect had been the result of TLR9 activation by CpG motifs present in the antisense molecules [309,315,316]. Polymorphisms in the TLR9 gene have been associated with HIV-1 disease progression. Two SNPs (1635A/G and +1174G/A) are found at higher frequency in HAART-naïve rapid progressors (estimated as CD4 count decline) than in normal progressors [317].

Further highlighting the influence of co-infecting pathogens, malaria episodes result in increased viral loads in HIV-infected individuals, a factor that may have influenced the spread of HIV in sub-Saharan Africa [318-320]. Likewise, a study of Kenyan pregnant women has shown that susceptibility to malaria is enhanced up to 2-fold in HIV-infected patients [321]. Interestingly, although malaria did not significantly affect the frequency of mother-to-child transmission according to this report, co-infection with helminth parasites was associated with increased risk of transmission by a mechanism likely involving activation of intrauterine lymphocytes [321]. Importantly, not all co-infecting pathogens increase HIV replication. Infections with GB virus, measles virus, *Orientia tsutsugamushi *and HTLV type 1 and 2 viruses are known to attenuate HIV infection [322]. Human herpesvirus 6 (HHV-6) and 7 (HHV-7) also suppress HIV replication. Interestingly, these viruses inhibit infection with R5 tropic strains, but minimally affect replication of X4 virus. HHV-6 interferes with HIV-1 by upregulating the CCR5 ligand RANTES [323]. Unlike HHV-6, HHV-7 exerts its effect by downregulating CD4, the cellular receptor shared by HHV-7 and HIV-1 [324]. The R5-specific effect of the inhibition of HIV-1 replication by HHV-7 may be explained by lower affinities for CD4 in R5 envelopes, as compared to T-tropic envelopes.

The FC gamma receptor IIa (CD32) has also been shown to influence HIV pathogenesis. This receptor is expressed in B cells and monocytes, and plays an important role in the removal of antibody-coated infectious agents [325]. One group has evaluated the effect of polymorphisms of the FC gamma receptor IIa gene in HIV-1 perinatal transmission in Kenyan women. The frequency of a His/His131 SNP is higher in HIV-positive than in HIV-negative children, whereas distribution of an Arg/Arg131 allele is reversed [326]. Another study with a Kenyan cohort found similar conclusions for the same alleles in transmission of placental malaria [327]. Unlike the His131 form, the Arg131 variant does not bind IgG2, however, further experimental evidence is needed to evaluate whether this property of the FC gamma receptor is responsible for the observed phenotype.

Cytotoxic T lymphocytes (CTL) play an essential role in the response to HIV infection. It is well accepted that the efficacy of the CTL responses vary among infected individuals and modulate disease progression in vivo [13,14]. In addition to HLA variants, other molecules modulate the efficacy of the CTL response. CTL-dependent killing of target cells is mediated by the content of storage granules released near the junction of the CTL and target cell. These granules contain the pore-forming protein perforin, and several serine proteases called granzymes [328]. HIV-specific CTLs from LTNP achieving immunological control proliferate extensively in response to autologous HIV-infected CD4-positive T cells. These LTNP CTLs, unlike those in normal progressors, express higher levels of perforin upon stimulation and proliferation with autologous cells [329]. This suggests that the quality of the CTL response plays an essential role in the response against HIV infection. Supporting this, polymorphisms in the perforin gene have been associated with slower disease progression in a French cohort. The presence of the SNP 1012T in the proximal perforin promoter correlates with fewer perforin-positive CTL cells, whereas homozygous individuals (1012T/T) display a trend towards slower disease progression [330].

## Conclusion

To date, considerable progress has been made in identifying allelic variations associated with AIDS progression and HIV-1 transmission. However, this work is beset by problems. These include the polygenic and multifactorial nature of the disease, and the difficulties in isolating the effect of individual allelic variations in genetically diverse cohorts of patients. The complexities of characterizing and recruiting individuals for LTNP and EU cohorts, and the genetic differences between ethnicities have also confounded the characterization of significant associations. Despite this, it is anticipated that in the near future, the map of genes involved in HIV-1 pathogenesis will be greatly enhanced. Identification of these genes and the variants associated with slow progression and resistance to infection will allow physicians to predict better the outcome of disease and to design antiretroviral regimes tailored to patient-specific genotypes. As it has occurred with the CCR5Δ32 allele, the discovery of new variants might invigorate the development of approaches to combat HIV-1 infection. Furthermore, interpretation of clinical data evaluating anti-HIV drugs and vaccines will also benefit from an understanding of the effects of genetic predisposition/resistance of the patients.

## Abbreviations

CTL, cytotoxic T lymphocyte; CypA, cyclophilin A; EU, highly exposed uninfected individuals; HHV, human herpesvirus; IDU, intravenous drug users; LTNP, long-term nonprogressor; MHC, major histocompatibility complex; MVB, multivesicular body; SNP, single-nucleotide polymorphism; TLR, toll-like receptor.

## Competing interests

The author(s) declare that they have no competing interests.

## Authors' contributions

JL and VP participating equally in revising the intellectual content and drafting the manuscript. Both authors read and approved the final manuscript.
